# The impact of climate change on skin cancer incidence: mechanisms, vulnerabilities, and mitigation strategies

**DOI:** 10.3389/fpubh.2025.1674975

**Published:** 2025-10-13

**Authors:** Lin Wang, Yuan Chi, Jing Li, Xingxing Yuan

**Affiliations:** ^1^Department of Dermatology, Fourth Affiliated Hospital of Heilongjiang University of Chinese Medicine, Harbin, China; ^2^Department of Internal Medicine, Heilongjiang Academy of Traditional Chinese Medicine, Harbin, China

**Keywords:** climate change, skin cancer, UV radiation, ozone depletion, sun protection

## Abstract

Climate change is increasingly recognized as a major public health challenge with wide-ranging effects on health conditions, including skin cancer. Rising global temperatures and heightened ultraviolet (UV) radiation intensity due to ozone depletion are contributing to a significant increase in skin cancer cases worldwide. This review explores the impact of altered UV radiation levels, behavioral shifts, and environmental factors on vulnerable populations in relation to the connection between climate change and rising skin cancer incidence. This relationship is further complicated by several paradoxes involving human behavior, ozone layer recovery, and socioeconomic factors. The discussion focuses on the mechanisms linking climate change to higher skin cancer rates, particularly the roles of UV radiation exposure, increased temperatures, and ozone layer depletion. These environmental changes disproportionately affect vulnerable groups, such as children, the older adults, and populations in high-risk geographic regions. To mitigate the growing burden of skin cancer associated with climate change, public health strategies including sun safety education, early detection programs, and international climate policies must be prioritized. Predicting skin cancer incidence rates depends on current and past sun protection behaviors and preventive measures. This review underscores the need for a coordinated global response to climate change and its impact on skin cancer, emphasizing prevention, early diagnosis, and effective treatment.

## 1 Introduction

Global climate change, driven by human activities, profoundly impacts ecosystems and human health, including altering disease patterns (1). Of particular urgency is the documented relationship between climate change and rising incidence rates of preventable cancers, most notably cutaneous melanoma and keratinocyte cancers (basal cell and squamous cell carcinoma (SCC)) (2). Skin cancer, one of the most common malignancies globally, primarily stems from excessive ultraviolet (UV) radiation exposure, which induces DNA damage in epidermal cells, particularly keratinocytes and melanocytes (3). Alarmingly, global temperature elevations correlate strongly with intensified solar UV radiation reaching the Earth’s surface, thereby amplifying skin cancer risks in UV-vulnerable regions such as equatorial zones and high-altitude areas (4).

Epidemiological data reveal a marked increase in skin cancer incidence across regions experiencing pronounced climatic alterations. For example, a longitudinal study in Scandinavia reported a > 4% annual increase in melanoma incidence over the past two decades, correlating with prolonged summer seasons and more frequent heatwaves (5). Warmer climates lead to more outdoor activities and lifestyle changes, which inadvertently increase exposure to higher UV levels (6). Regions like Scandinavia and northern Canada, historically protected by low traditional sun exposure profiles such as shorter summers and lower UV indices, now report rising cases as climate patterns, specifically, polar-amplified warming and shifting jet streams alter these patterns, leading to extended periods of high solar irradiance (7). This mini-review examines the multifaceted relationship between climate change and skin cancer epidemiology, focusing on the mechanisms of UV radiation, ozone depletion, and temperature-induced behavioral changes. Furthermore, it highlights disproportionately affected high-risk groups and synthesizes current evidence to inform targeted prevention strategies and adaptive public health policies.

## 2 Effects of climate change factors on skin cancer

UV radiation induces direct DNA damage through the formation of cyclobutane pyrimidine dimers (CPDs) and 6–4 photoproducts, which, if not adequately repaired, lead to characteristic UV-signature mutations such as C → T and CC → TT transitions. These mutations drive cutaneous carcinogenesis by disrupting key oncogenes and tumor suppressor genes, resulting primarily in basal cell carcinoma (BCC), SCC, and melanoma (8, 9). While BCC typically exhibits slow growth and local invasiveness, SCC possesses a greater potential for metastasis if left untreated. Melanoma, although less common, is responsible for the majority of skin cancer-related mortality due to its highly aggressive behavior and propensity for early dissemination (10, 11).

### 2.1 Increased ultraviolet radiation

The relationship between UV exposure and skin cancer is complex, involving various factors such as the intensity and duration of exposure, geographical location, and individual susceptibility. While climate change does not directly generate UV radiation, it exacerbates exposure through indirect mechanisms. Warmer atmospheric conditions reduce UV absorption capacity, particularly in equatorial regions, allowing more solar radiation to reach terrestrial surfaces (12). Concurrently, shifting weather patterns prolong sunny periods, such as extended summer months (March to October in temperate regions) with earlier sunrises and later sunsets, while higher temperatures (consistently exceeding 25 °C) encourage prolonged outdoor activities during peak daylight hours, both significantly extending UV exposure duration (13). A review on UV-induced immunosuppression discusses how UV exposure can down-regulate immune responses, thereby increasing the risk of skin cancer. UV radiation induces localized and systemic immunosuppression by modulating cytokine production and activating regulatory T cells, which diminishes the immune system’s ability to recognize and eliminate UV-damaged cells, thereby increasing skin cancer risk (14). Furthermore, the role of UV radiation in skin carcinogenesis is underscored by its ability to cause genetic mutations and promote the unregulated proliferation of skin cells, as discussed in a comprehensive review on the subject. UV radiation induces signature mutations, such as C → T and CC → TT transitions, primarily in tumor suppressor genes like *TP53* in squamous cell carcinoma and *PTCH1* in basal cell carcinoma, which disrupt apoptosis and promote uncontrolled cellular proliferation, as detailed in a comprehensive review on UV-induced skin carcinogenesis (15). The Stockholm public health cohort study explored the association between skin cancer and various UVR indicators finding significant associations with skin cancer risk (16). These findings underscore the multifaceted nature of UV radiation’s impact on skin cancer development and highlight the need for effective prevention strategies, including public education and behavioral modifications to reduce UV exposure.

### 2.2 Stratospheric ozone depletion

The Montreal Protocol, an international treaty signed by 197 countries, has been instrumental in phasing out the production of ozone-depleting substances. This treaty has not only contributed to the gradual recovery of the ozone layer, a projected return of mid-latitude and polar ozone levels to 1980 values by the mid-21st century but also played a role in mitigating climate change, as many of the substances controlled under the protocol (chlorofluorocarbons (CFCs) and hydrochlorofluorocarbons (HCFCs)) are also potent greenhouse gases with a global warming potential thousands of times greater than carbon dioxide. The stratospheric ozone layer, absorbing ~90% of harmful UV radiation, has been critically degraded by CFCs despite the Montreal Protocol’s regulatory success (17–19). Persistent ozone holes over polar regions permit heightened UV penetration, disproportionately affecting sparsely populated Arctic/Antarctic zones and mid-latitude countries. However, the path to ozone recovery is subject to a complex paradox where climate change can alter stratospheric temperatures and wind patterns, potentially delaying the restoration of protective ozone layers in some regions and prolonging elevated UV exposure risks. Australia exemplifies this crisis, where ozone depletion synergizes with geographic factors to yield the world’s highest skin cancer prevalence (20, 21). Notably, delayed ozone layer recovery prolongs UV exposure even in temperate regions, escalating risks for populations unadapted to intense solar radiation (22).

### 2.3 Global warming and altered ultraviolet radiation patterns

The interactive effects of stratospheric ozone depletion and climate change further complicate the scenario. Climate change can influence the dynamics of ozone depletion, while ozone depletion itself can affect climate patterns. These interlinking effects have implications for air quality, ecosystems, and human health. The Environmental Effects Assessment Panel of the Montreal Protocol has been evaluating these complex interactions, emphasizing the importance of continued monitoring and research to understand the full scope of these environmental changes (4).

Global warming has become an important environmental issue, reshaping the distribution of ultraviolet radiation through disrupted weather systems. Extended summers and intensified heatwaves prolong high-UV exposure windows, while orbital and atmospheric alterations redistribute solar radiation geographically (23, 24). Urban heat islands, intensified by climate change, further elevate risks in cities through dual temperature and UV spikes (25). Moreover, the interaction between increased environmental temperatures and UV radiation may further complicate the risk factors for skin cancer. Elevated temperatures can enhance the effects of UV radiation on the skin, accelerating photoaging and increasing the likelihood of photocarcinogenesis. Studies have shown that higher environmental temperatures can influence the biological effects of UV radiation, potentially leading to more severe skin damage, such as deep tissue inflammation and accelerated photoaging, and a higher incidence of skin cancer (26).

### 2.4 Shifting population demographics and increased migration

As climate change progresses, it can lead to shifts in population distributions due to factors such as rising sea levels, increased frequency of extreme weather events, and changes in agricultural productivity. These shifts can, in turn, affect the exposure of populations to UV radiation, a major risk factor for skin cancer. Migrants from low-UV regions like northern latitudes to sun-intensive areas often lack protective behaviors like sunscreen use, increasing susceptibility to burns and cumulative damage (27). This adaptation gap, combined with limited awareness, drives abrupt rises in skin cancer incidence among relocated groups, highlighting the need for targeted education in migration corridors.

### 2.5 Climate change and behavioral shifts

Climate change fundamentally reshapes skin cancer epidemiology. Rising melanoma and non-melanoma skin cancer rates to elevated temperatures, extended summer seasons, and heightened UV radiation exposure (28, 29). Notably, geographic patterns of skin cancer risk are shifting—regions like Scandinavia and parts of Canada, traditionally considered low-risk due to limited sun exposure, now report increasing cases as populations face unfamiliar UV intensity levels. A seminal European study found melanoma spikes correlate strongly with increased solar radiation and unusually high summer temperatures, revealing new vulnerabilities in cooler climate populations (30). These studies highlighted the importance of understanding how climate change can influence human behavior, leading to increased health risks such as skin cancer.

Warmer climates promote outdoor lifestyles, inadvertently increasing UV exposure. Leisure activities such as hiking, cycling, and tourism along with urban recreation surge with rising temperatures, yet sun protection practices lag behind behavioral changes (31, 32). Northern populations, previously sheltered by colder climates, now engage in prolonged outdoor activities without established protective habits, accelerating photoaging and carcinogenesis (33, 34). Tourism exacerbates risks, as high-altitude destinations with thinner atmospheres and reflective snowscapes intensify UV damage, often overlooked by underprepared visitors (35–37).

Furthermore, public health efforts face a significant behavioral paradox (28) where climate change creates more pleasant and inviting weather that encourages extended outdoor activities, inadvertently increasing population-wide UV exposure. This creates a counterintuitive scenario where improved weather conditions, a perceived benefit of climate change, can ultimately lead to adverse health outcomes by undermining sun-protection messages. Effective public health campaigns must therefore navigate this paradox by promoting sun-safe practices within the context of an active outdoor lifestyle, rather than simply advising against outdoor activities.

### 2.6 The complex role of air pollution in ultraviolet exposure

**Figure 1:**
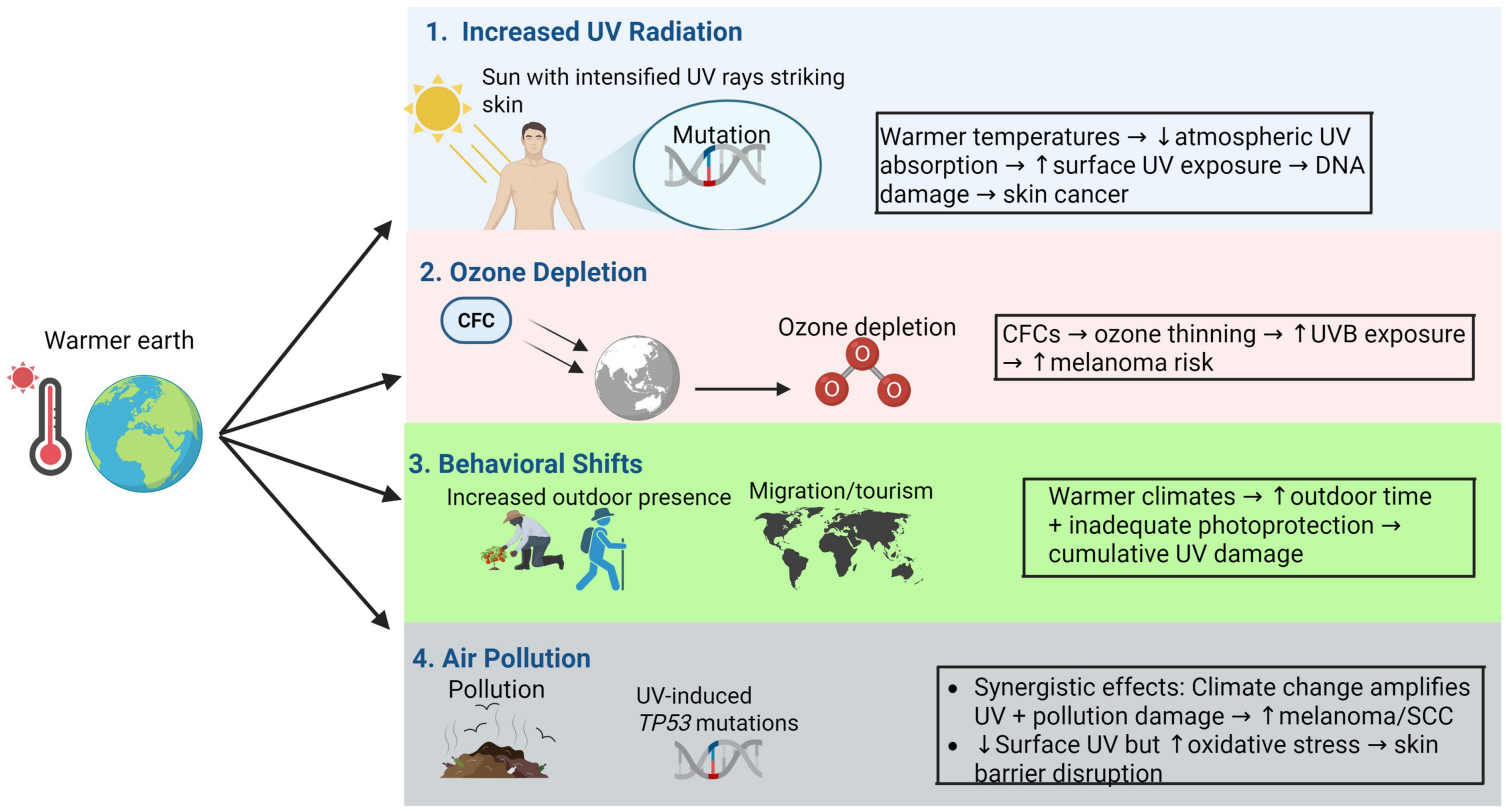
Effects of climate change factors on skin cancer.

Climate change paradoxically modulates UV-skin interactions through air pollution. While particulates scatter UV radiation, reducing surface levels, pollutants like black carbon and ozone directly damage skin via oxidative stress and inflammation (38, 39). Chronic exposure impairs collagen synthesis and epidermal integrity, synergizing with UV to elevate urban skin cancer rates (40). Wildfire-driven pollution surges, intensified by climate change, further threaten cities where heat islands concentrate both pollutants and outdoor activities (41). This dual exposure creates a novel risk axis, demanding integrated mitigation strategies (Figure 1).

## 3 New research findings on the link between climate change and skin cancer

### 3.1 Innovative sun protection technologies

While climate change amplifies UV exposure risks, parallel technological breakthroughs are redefining photoprotection strategies. The effectiveness of sun protection methods, including the use of sunscreens, protective clothing, and behavioral changes, has been the subject of extensive research.

#### 3.1.1 Next-generation sunscreens

Next-generation sunscreens represent significant advancements over traditional formulations, offering superior, longer-lasting protection characterized by high efficacy, providing SPF 50 + with broad-spectrum (UVA/UVB) coverage, enhanced durability, such as 8-h photostability and water resistance to improve compliance during prolonged outdoor exposure, and improved formulations that incorporate advanced filters (Tinosorb S, Mexoryl XL) and antioxidant complexes (vitamin C, ferulic acid) to counteract oxidative stress and deliver more comprehensive protection against photodamage.

#### 3.1.2 Advanced textiles and wearable sensors

Sun-protective clothing with ultraviolet protection factors (UPF) exceeding 50 offers reliable, chemical-free protection. Wearable UV sensors provide real-time, personalized exposure monitoring, alerting users to potential overexposure risks and empowering informed behavioral modifications (42–44). Sunscreens are a widely used form of sun protection, and their effectiveness in preventing skin cancer has been supported by evidence from randomized controlled trials. For instance, a study conducted in Australia demonstrated that the regular application of broad-spectrum sunscreen could reduce the risk of developing cutaneous SCC and melanoma, although it did not significantly impact the incidence of BCC. This finding underscores the importance of using sunscreen as part of a comprehensive sun protection strategy (45).

### 3.2 Artificial intelligence and early detection

Artificial intelligence (AI) and machine learning are transforming dermatology through enhanced skin cancer detection capabilities. These technologies enable earlier and more accurate identification of malignant lesions, particularly valuable in regions with limited access to dermatologists (46). AI powered tools allow users to self-monitor suspicious skin changes, providing early warnings of potential malignancies. Developing predictive algorithms that assess skin cancer risk by analyzing genetic predispositions alongside lifestyle and environmental exposure factors is being implemented (32, 47). This approach promises more personalized prevention strategies and targeted screening programs, ensuring high-risk individuals receive timely interventions and education about their specific risk profiles.

### 3.3 Emerging skin cancer treatment options

Immunotherapy, particularly checkpoint inhibitors, has revolutionized melanoma treatment, improving survival rates for advanced-stage patients (48). Targeted therapies attack specific genetic mutations, offering precision over traditional chemotherapy. Photodynamic therapy (PDT) shows particular promise for treating various skin cancers (49). This approach combines light-activated photosensitizing agents with targeted illumination to selectively destroy malignant cells while sparing healthy tissue. Current research explores expanding PDT applications from superficial tumors to deeper or larger malignancies.

## 4 Interventions to mitigate climate impact on skin cancer-taking references from case studies

While skin cancer is largely preventable through sun safety measures, climate change may exacerbate UV exposure risks, necessitating enhanced prevention and early detection strategies (50). To address these challenges, targeted interventions are essential, particularly for high-risk populations and regions facing unique environmental and socioeconomic vulnerabilities. Certain populations face elevated vulnerability to climate change-driven skin cancer risks. These include residents of high-UV regions (equatorial zones, high-altitude areas) and socioeconomically disadvantaged groups with limited access to healthcare, education, and protective resources (sunscreen, shade) (51). Prolonged sunlight exposure due to rising temperatures further increases UV risks in these regions (52). Low-income communities often lack healthcare access, delaying skin cancer screenings and worsening outcomes. Age also influences susceptibility: children’s sensitive skin and outdoor activity increase damage risk, while the older adults accumulate lifetime UV damage (53). Migrant populations relocating from low- to high-UV areas face heightened sunburn risk due to unfamiliarity with sun protection.

Australia exemplifies climate change’s impact on skin cancer. Its high baseline UV exposure has intensified due to ozone layer depletion from CFCs (54). With the world’s highest skin cancer incidence (affecting ~50% of Australians), public health strategies like the SunSmart campaign (since 1981) promote sunscreen use, protective clothing, and shade-seeking during peak UV hours (55). Despite reducing melanoma rates in younger cohorts, climate change prolongs UV exposure, challenging prevention efforts. Moreover, northern countries (Sweden, Finland) report increasing skin cancer rates linked to warmer summers and extended UV exposure (56). Previously low-risk due to cooler climates, these regions now face heightened risk from heatwaves and cultural shifts toward sunbathing. Public health campaigns emphasize sunscreen use and screenings, targeting fair-skinned individuals and those with family histories (57). In addition, Urban heat islands (elevated temperatures from infrastructure and scarce greenery) amplify UV exposure in cities like Los Angeles and New York (58). Outdoor workers (construction crews) face reflected UV radiation, while tanning bed use persists despite known risks. Air pollution exacerbates skin damage via free radical formation (59). Mitigation includes sun safety education, free screenings, and urban green spaces (60).

These case studies demonstrate that the impact of climate change on skin cancer is profoundly mediated by a socioeconomic paradox (61). Lower socioeconomic status populations often have less recreational sun exposure but experience higher rates of occupational UV exposure and later-stage skin cancer diagnoses. This disparity stems from barriers such as lack of access to sunscreen, protective clothing, shade, and healthcare, as well as less sun safety education. This paradox was exacerbated during the COVID-19 pandemic, where reduced healthcare access disproportionately affected these groups. As climate change increases UV exposure risks, it will likely widen these existing health inequities, as vulnerable populations have the fewest resources to adapt to environmental changes. These cases highlight the need for region-specific strategies to address unique local challenges and reduce UV radiation and pollutant exposure, mitigating future global skin cancer burdens.

## 5 Methods of reducing skin cancer incidence resulting from climate change

Public health experts and policymakers must address climate change’s contribution to rising skin cancer incidence. While large-scale environmental changes require systemic action, effective strategies exist to mitigate risk, promote prevention, and improve outcomes. Key approaches include public health campaigns, international policy actions, and research innovations in detection and treatment.

### 5.1 Public health campaigns and education

Public health campaigns emphasizing UV radiation dangers and sun protection are crucial. Promoting sun-safe behaviors like sunscreen use, protective clothing, and seeking shade during peak sunlight is essential, especially as climate change increases temperatures and sunlight exposure. Global and national health organizations, such as the WHO, should implement tailored campaigns for high-risk groups like children, the older adults, and outdoor workers. Messages should be disseminated through TV, social media, and community programs, addressing risks from tanning beds. Schools, summer camps, and health centers play a vital role in educating families about sun protection. Employers should provide free or subsidized sunscreen and protective gear for outdoor workers and incorporate sun safety into workplace wellness programs (62).

### 5.2 Policy actions and international cooperation

International cooperation and policy changes are essential to address climate change and reduce UV radiation exposure. Policies targeting carbon emissions, ozone layer protection, and environmental degradation can mitigate skin cancer risks (63). The Montreal Protocol, which reduced ozone-depleting CFCs, remains a key agreement. Continued ozone layer protection and efforts to reduce greenhouse gas emissions are critical (64). Governments should promote renewable energy, improve air quality, and implement urban design strategies like shaded areas and green roofs to reduce sunlight exposure (65, 66). At the national level, policies should target high-risk populations, such as those living in regions with high UV exposure, by improving access to skin cancer prevention tools and early detection services. These policies should target high-risk populations by improving access to prevention tools and early detection services, such as subsidized sunscreen and free screenings. Collaboration with healthcare providers is necessary to ensure culturally appropriate materials for migrant populations.

### 5.3 Research and innovation in skin cancer detection and treatment

**Figure 2:**
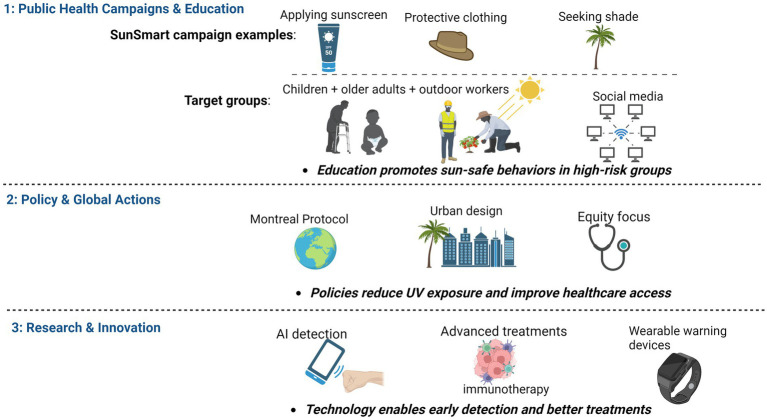
Strategies to reduce climate change-driven skin cancer risk.

Research and innovation in early detection, treatment, and prevention are vital to reduce the impact of climate change on skin cancer. Advances in diagnostic tools and treatment methods are needed to address rising incidence (67). Technologies like dermoscopy and AI-powered mobile applications improve skin cancer detection, enabling early medical intervention (68). Routine screenings, particularly for vulnerable communities, enhance early diagnosis and treatment outcomes (69). Treatment options, such as immunotherapy and targeted therapies, have advanced significantly, improving outcomes for melanoma patients. Research must prioritize developing more effective treatments and ensuring global access to these technologies. Innovations in preventive technologies, like sun-protective fabrics and wearable UV monitors, can improve sun safety practices (Figure 2).

## 6 Conclusion

Climate change is intensifying skin cancer risks, requiring a multifaceted response. Rising temperatures and UV levels are increasing skin cancer incidence, even in previously low-risk regions. While advancements in diagnostics, sun protection, and treatments are promising, they are insufficient alone. Comprehensive public health strategies are crucial, including sun safety education, climate action to curb UV exposure, and better access to early detection and care. Factors like ozone depletion, shifting UV patterns, and climate-driven migration further complicate the issue. This review discusses public health campaigns and education as key mitigation strategies. This paradox explains why such campaigns are necessary yet often challenging, they must motivate individuals to act for a collective, statistical good. This review directly addresses this in sections on behavioral shifts and migration, describing how warmer climates promote outdoor lifestyles and how migrants may lack protective behaviors. The paradox lies in the fact that the appealing weather created by climate change counterintuitively increases the risk it necessitates protecting against. Collaboration among public health officials, policymakers, and researchers is essential to protect vulnerable populations and reduce the skin cancer burden. Integrating prevention into global health initiatives is urgent. Through innovation, policy reform, and education, we can mitigate climate change’s health impacts and strive for a future with reduced cancer risks and improved public health outcomes.
